# Environmental and social benefits of the targeted intraoperative radiotherapy for breast cancer: data from UK TARGIT-A trial centres and two UK NHS hospitals offering TARGIT IORT

**DOI:** 10.1136/bmjopen-2015-010703

**Published:** 2016-05-03

**Authors:** Nathan J Coombs, Joel M Coombs, Uma J Vaidya, Julian Singer, Max Bulsara, Jeffrey S Tobias, Frederik Wenz, David J Joseph, Douglas A Brown, Richard Rainsbury, Tim Davidson, Douglas J A Adamson, Samuele Massarut, David Morgan, Ingrid Potyka, Tammy Corica, Mary Falzon, Norman Williams, Michael Baum, Jayant S Vaidya

**Affiliations:** 1 Department of Breast Surgery, Great Western Hospital, UK; 2 Royal Wootton Bassett Academy, UK; 3 Nonsuch High School for Girls, UK; 4 Surgical and Interventional Trials Unit of the Division of Surgery and Interventional Science, University College London, London, UK; 5 Department of Radiotherapy, Princess Alexandra Hospital, Harlow, UK; 6 University of Notre Dame, Fremantle, Western Australia, Australia; 7 Department of Radiation Oncology(JST), University College London, London, UK; 8 Department of Radiation Oncology, University of Heidelberg, Germany; 9 Department of Radiation Oncology, Sir Charles Gairdner Hospital, Nedlands, Western Australia, Australia; 10 Ninewells Hospital, Dundee, UK; 11 Royal Hampshire County Hospital, Winchester, UK; 12 Royal Free Hospital, London, UK; 13 Centro di Riferimento Oncologica, Aviano, Italy; 14 Sherwood Forest Hospitals NHS Foundation Trust, Nottinghamshire, UK; 15 Department of Pathology, University College London, London, UK

**Keywords:** Breast cancer, RADIOTHERAPY, TARGIT, IORT, targeted intraoperative radiotherapy

## Abstract

**Objective:**

To quantify the journeys and CO_2_ emissions if women with breast cancer are treated with risk-adapted single-dose targeted intraoperative radiotherapy (TARGIT) rather than several weeks' course of external beam whole breast radiotherapy (EBRT) treatment.

**Setting:**

(1) TARGIT-A randomised clinical trial (ISRCTN34086741) which compared TARGIT with traditional EBRT and found similar breast cancer control, particularly when TARGIT was given simultaneously with lumpectomy, (2) 2 additional UK centres offering TARGIT.

**Participants:**

485 UK patients (249 TARGIT, 236 EBRT) in the prepathology stratum of TARGIT-A trial (where randomisation occurred before lumpectomy and TARGIT was delivered simultaneously with lumpectomy) for whom geographical data were available and 22 patients treated with TARGIT after completion of the TARGIT-A trial in 2 additional UK breast centres.

**Outcome measures:**

The shortest total journey distance, time and CO_2_ emissions from home to hospital to receive all the fractions of radiotherapy.

**Methods:**

Distances, time and CO_2_ emissions were calculated using Google Maps and assuming a fuel efficiency of 40 mpg. The groups were compared using the Student t test with unequal variance and the non-parametric Wilcoxon rank-sum (Mann-Whitney) test.

**Results:**

TARGIT patients travelled significantly fewer miles: TARGIT 21 681, mean 87.1 (SE 19.1) versus EBRT 92 591, mean 392.3 (SE 30.2); had lower CO_2_ emissions 24.7 kg (SE 5.4) vs 111 kg (SE 8.6) and spent less time travelling: 3 h (SE 0.53) vs 14 h (SE 0.76), all p<0.0001. Patients treated with TARGIT in 2 hospitals in semirural locations were spared much longer journeys (753 miles, 30 h, 215 kg CO_2_ per patient).

**Conclusions:**

The use of TARGIT intraoperative radiotherapy for eligible patients with breast cancer significantly reduces their journeys for treatment and has environmental benefits. If widely available, 5 million miles (8 000 000 km) of travel, 170 000 woman-hours and 1200 tonnes of CO_2_ (a forest of 100 hectares) will be saved annually in the UK.

**Trial registration number:**

ISRCTN34086741; Post-results.

**Strengths and limitations of this study**

- This study calculated journeys made by patients with breast cancer to receive their radiotherapy, using the geographic and treatment data from a large randomised trial.
- The study then assessed the same outcomes (travel distances, travel time and CO_2_ emissions) in two semirural breast cancers—the results of this assessment confirm and reinforce the original results: the benefit of the use of TARGIT for patients from two semirural breast centres was even larger (753 miles (1212 km), 30 h, 215 kg CO_2_ per patient).
- The carbon emissions were calculated from measured fuel economies in a standard family car in real-world driving conditions rather than relying on a car manufacturer's claimed emission figures that are derived under strict test conditions.
- Although the patients' addresses and address of the radiotherapy centre were known and used to calculate the journey, the exact daily travel for each patient was not available. However, this is unlikely to affect the results. Similarly, our estimates for carbon emissions assume a standard family car, so it may not be exact.

## Introduction

Awareness of the impact of climate change has led to increasing information being displayed about the carbon footprint of certain activities. For example, we are now more aware of the concept of ‘food-miles’ and the benefits of buying locally sourced products. With increasing centralisation of resources in healthcare, the trend is exactly the reverse of the trend to ‘shop locally’. There is in fact a greater demand on patients and their families to travel to receive specialist treatment.^1^ ^2^

Numerous studies have assessed the impact of travel time and distance on a patient receiving healthcare or choosing treatment options.^3^ ^4^ In some international studies, patients who had to travel many miles for radiotherapy after breast cancer treatment chose a mastectomy rather than breast conservation^5–7^ and multiple fractions of whole breast radiotherapy, but this was not seen in some UK studies.^3^ ^8^ Similarly, the uptake of chemotherapy or postmastectomy radiotherapy may be lower in rural communities where travel to a radiotherapy centre is difficult.^9^ ^10^ The daily travel for patients and their relatives will often affect their quality of life and impact on them and their family members.^1^ ^2^

The management of breast cancer has changed over the decades. However, the requirement of patients to travel to receive these specialist services is often forgotten by policymakers.^11^ Conventionally, patients who have breast cancer and breast-conserving surgery are recommended to receive whole breast external beam radiotherapy (EBRT) daily, over 3–6 weeks following surgery.

**Figure 1:**
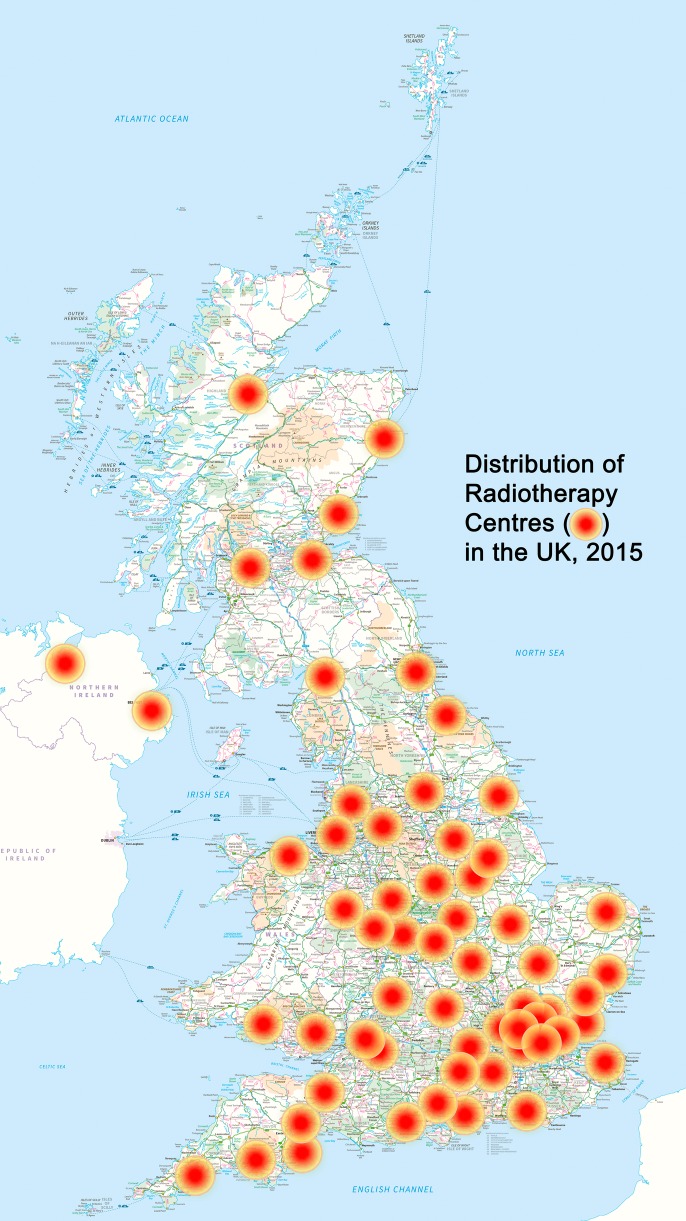
Map of the UK showing the locations of radiotherapy centres with a radius of 13 miles (20 km) drawn around them. Two-thirds (63%) of the UK population live outside of towns that have a radiotherapy centre (data given in online supplementary table S2). Contains OS data © Crown copyright 2016, and reference 28.

Current UK provision of radiotherapy within the National Health Service (NHS) is based in 62 hospital sites (figure 1): England—52, Scotland—5, Wales—3 and Northern Ireland—2.^12^ The National Radiotherapy Advisory Group recommends that travel times should be less than 45 min for the majority of patients as this is known to impact on access and uptake.^13^ The red dots in figure 1 show a radius of 13 miles (21 km), which is the average distance of a patient from the radiotherapy centre in the TARGIT-A trial, thus showing how large areas remain outside these perimeters. Accounting for the population density, we have estimated that two-thirds of the UK population lives more than 13 miles from a radiotherapy centre (figure 1 and supplementary table 2).^28^

10.1136/bmjopen-2015-010703.supp1 Supplementary tables

Approaches such as the use of risk-adapted targeted intraoperative radiotherapy (TARGIT IORT)^14^ ^15^ can obviate the need for whole breast EBRT in selected patients.^16^ ^17^ Indeed, single-dose TARGIT IORT is now offered routinely as a treatment option in many other countries.^18^

The TARGIT-A trial was an international randomised controlled trial initiated in the UK that showed that a single dose of IORT using the Intrabeam device (Carl Zeiss) was not inferior to traditional EBRT in local control after breast-conserving surgery.^17^ This delivers a single fraction of radiotherapy (20 gy) into the tumour cavity and adds about 20–40 min to the operative procedure. UK centres contributed 20% of the 3451 patients recruited in the TARGIT-A trial from 33 centres in 11 countries worldwide.

This study compares the travel implications and journey times within each randomised treatment group of the TARGIT-A trial in the UK.

We also measured the impact of introducing TARGIT IORT in two UK breast centres without on-site radiotherapy and assessed the likely environmental gains to be made by implementing TARGIT IORT in the management of early breast cancer in the UK.

## Methods

### TARGIT-A trial data

Geographic and radiotherapy data from the UK patients who had been recruited into the TARGIT-A randomised controlled trial were assessed. In six UK hospitals (University College London, Royal Free, Whittington, Guy's and St Thomas' (all in London), Ninewells (in Dundee, Scotland) and Royal Hampshire County (in Winchester)), the patients undergoing breast-conserving surgery either received traditional EBRT or were selected to receive TARGIT IORT as a single dose using the Intrabeam device (Carl Zeiss). Patients who received TARGIT were recommended additional breast EBRT (without a tumour bed boost) if their final tumour histology had prespecified adverse prognostic factors. Fifteen per cent to 20% of patients randomly allocated to receive TARGIT were expected to receive additional EBRT. Supported by the results of the TARGIT-A trial,^17^ the preferred method of using TARGIT is during initial lumpectomy, and therefore, for this paper, we restricted the analysis to the prepathology stratum of the TARGIT-A trial.

For each patient, we first calculated the shortest driving distance from home to the radiotherapy centre and travel time (excluding traffic delays) using Google Maps. We then calculated the total distance travelled and total journey time to receive all of the recorded fractions of radiotherapy for each patient. We assumed that patients who received EBRT required two additional journeys, for consent and for radiotherapy planning. Typically, a patient receiving 15 fractions of EBRT (3 weeks of radiotherapy) would attend the radiotherapy centre on at least 17 occasions. Amongst patients selected to have EBRT, those who lived a considerable distance from a TARGIT trial centre (more than 60 miles, 100 km) were excluded from the analysis (n=7) as they may have chosen to travel to a local radiotherapy centre closer to their home. A comparison was made between the aggregate distance and travel times between the two treatment arms (TARGIT vs EBRT) and between TARGIT-A trial centres in London, Winchester and Dundee.

### Swindon and Harlow patients

In 2014, two UK breast centres without on-site radiotherapy units (in Swindon and Harlow) started using TARGIT IORT. Using the patient's postcode and Google Maps, the distance that each of the 22 patients would have driven to their local radiotherapy centres (Oxford, Bath, North Middlesex or Cambridge) was calculated.

To assess the impact of travelling to a radiotherapy centre from his own hospital, the first author (NJC) undertook six return journeys from Swindon to Oxford and from Swindon to Bath using a medium-sized family car (a 7-year-old car with a 1.9 L diesel engine) in normal driving conditions, during a weekday and outside of peak times, and measured the actual distance travelled, time taken and fuel used. The estimates using Google maps were found to be an accurate reflection of such journeys (see online supplementary table S1). Therefore, for each patient with breast cancer treated with lumpectomy and TARGIT, we could estimate these values for travelling between their home and the radiotherapy centre using Google maps. We estimated the total travel distance assuming a standard 3-week course of radiation for the 22 patients who received TARGIT IORT mainly as part of training for participation in the TARGIT-B trial (http://www.nets.nihr.ac.uk/projects/hta/1010407, http://goo.gl/sgdcTr) in the past 15 months.

### Estimation of CO_2_ emission

We estimated the carbon dioxide produced by private transport based on the following measurement and assumptions: the fuel economy of the car was 39.7 miles per gallon (mpg; 6.96l/100 km), public transport usage was negligible, and half of the cars used diesel as a fuel and half used petrol. The CO_2_ produced by a 40 mpg diesel car is 299 g/mile (186 g/km) and that produced by a 40 mpg petrol car is 272 g/mile (169 g/km).^19^

### Statistical analysis

The null hypothesis was no difference in travelling distance or time between the two randomised groups. For statistical analyses, given that the distances travelled were skewed (not normally distributed) for at least one of the randomisation arms (TARGIT), we used both the Student t test with unequal variance as well as the non-parametric Wilcoxon rank-sum (Mann-Whitney) test. We used Microsoft Excel and STATA V.14.0 for statistical analysis.

## Results

### TARGIT-A trial data

Between 1999 and 2012, 714 patients were recruited to the UK centres for the TARGIT-A trial, and of these, 568 were in the prepathology stratum. Those patients randomised to receive TARGIT had their radiotherapy at the time of their primary surgery. Eighty-three patients (TARGIT 50, EBRT 33) were excluded from analysis due to insufficient (n=70) or inaccurate (n=6) home postcode details, or where the patient would have travelled to a closer radiotherapy centre to receive EBRT (n=7), leaving 485 (85.4%) for data analysis. Of these, 236 patients (48.7%) had been randomised to receive EBRT. In the 249 patients who had been randomised to receive TARGIT, 46 (18.5%) received additional EBRT.

#### Travel distance

**Figure 2:**
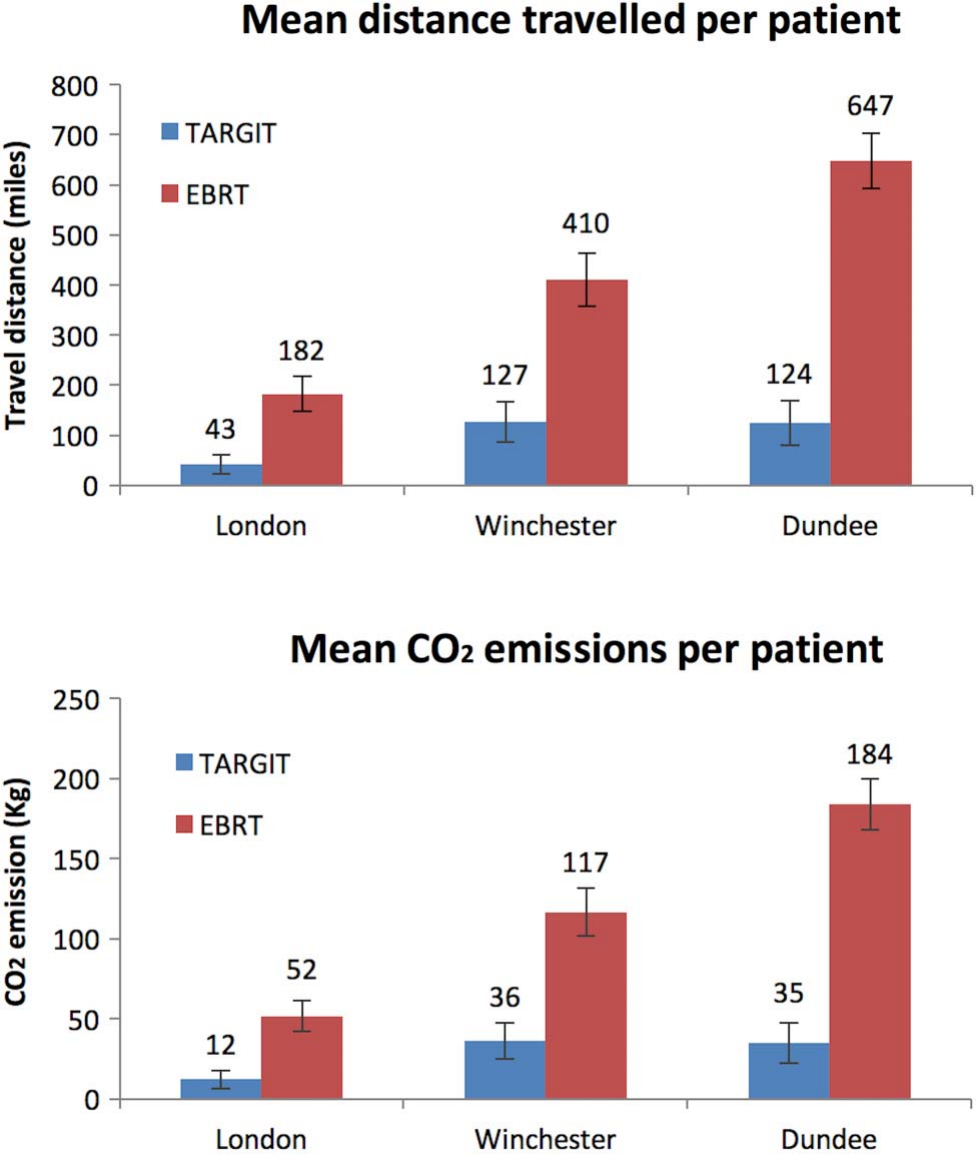
The mean distance travelled (above) and CO_2_ emissions (below) per patient for the allocated treatment. The error bars show the SE of the mean. (1 mile=1.61 km). EBRT, external beam whole breast radiotherapy.

Overall, these 485 UK patients would have travelled 114 273 miles (183 905 km; TARGIT 21 681 (34 892 km) versus EBRT 92 591 (149 011 km)) for planning, consent and receiving radiotherapy as part of the TARGIT-A trial, with those in the TARGIT arm travelling considerably less than those in the EBRT arm (mean distance driven in miles: TARGIT 87.1 (SE 19.1) versus EBRT 392.3 (SE 30.2), in kilometres: TARGIT 140.2 (SE 30.7) versus EBRT 631.4 (SE 48.6), p<0.0001 Wilcoxon rank-sum test, and p<0.0001 with the Student t test assuming unequal variance). Thus, the patients in the TARGIT arm were saved, on average, a travel distance of 305.2 miles (SE 35.8; 491.2 km (SE 57.6)). The difference in travelling distance was more pronounced for patients in Dundee (TARGIT 123.9 miles (SE 44.2) vs EBRT 647.4 miles (SE 55.2), TARGIT 199.4 km (SE 71.1) vs EBRT 1041.9 km (SE 88.8)), reflecting its rural surroundings where, on average, each patient saved themselves a journey of 523.5 miles (SE 70.7), 842.5 km (SE 113.8; figure 2 top)

#### CO_2_ emission

We estimate the total CO_2_ emissions by UK patients in the prepathology stratum of the TARGIT-A trial to be 32.5 tonnes, of which 81% (26.3 tonnes) was contributed by patients in the EBRT arm. The TARGIT arm contributed 19% of the total CO_2_ (6.2 tonnes), which corresponds to the fact that 18.5% of these patients received additional EBRT. The mean CO_2_ emissions for each patient in the EBRT arm was 111.4 kg (SE 8.6), whereas the mean emission by those randomised to TARGIT was 24.7 kg (SE 5.4), p<0.0001; a reduction of 86.7 kg (SE 10.2) per patient. A much larger reduction in emissions by being in the TARGIT arm was seen in the patients treated in Dundee, reflecting the greater distance travelled by these patients. The mean CO_2_ emission for Dundee patients randomised to receive TARGIT was 35 kg (SE 12.5) compared with 184 kg (SE 15.7) for EBRT patients, a saving of 149 kg (SE 20.1) of CO_2_ (figure 2 bottom).

#### Travel time

**Figure 3:**
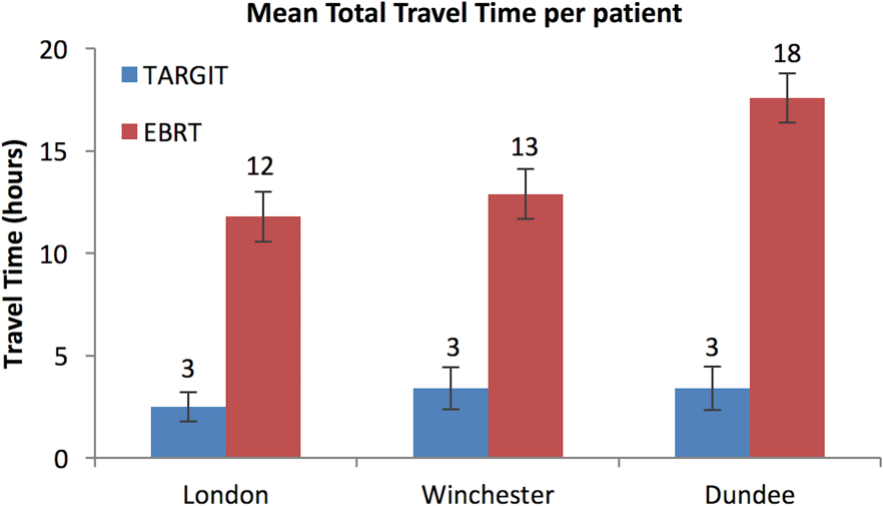
Mean time travelled by a patient for each allocated treatment. EBRT, external beam whole breast radiotherapy.

Overall, the mean time taken to travel for radiotherapy was 3.0 h (SE 0.53) for those randomised to TARGIT versus 14.0 h (SE 0.76) for EBRT, an average saving of 11 h (SE 0.92). The saving was longer for Dundee patients, at 14.2 h (SE 1.6). This does not include time spent in traffic jams, finding a parking space, waiting for the turn to receive the radiation dose, or actually receiving the fraction of radiotherapy. Of the 249 patients in the TARGIT arm, 81.5% (n=203) patients had received TARGIT IORT during lumpectomy and required no further travel for radiotherapy. The 46 patients who received additional EBRT travelled similar distances with similar journey times as those receiving traditional EBRT. Figure 3 shows the time taken to travel for radiotherapy in the three cities. Note that although the distances in London were shorter (figure 2), the time for travel for EBRT was relatively longer because of lower average speeds achievable in the city.

### Swindon and Harlow patients

**Figure 4:**
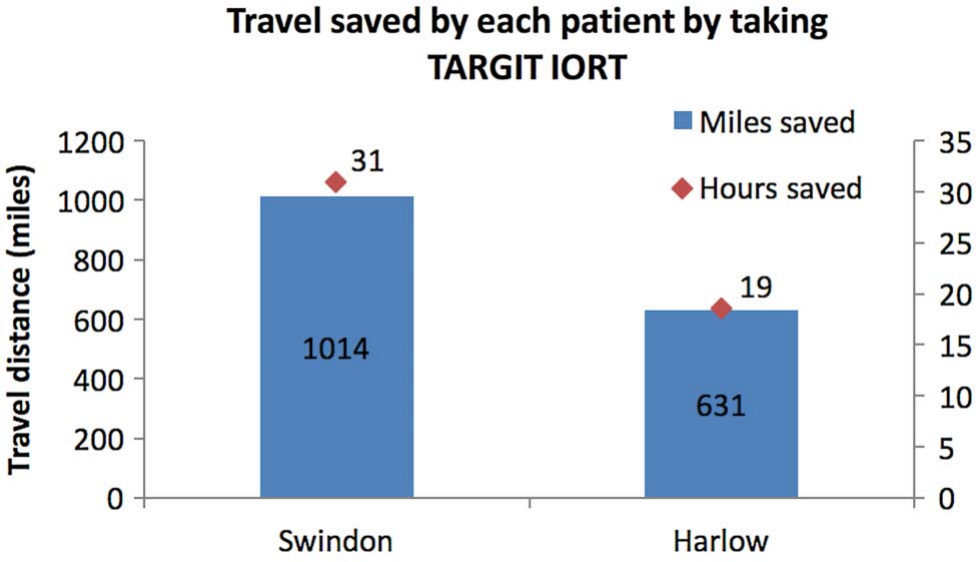
Estimated travel that was saved by patients in Swindon and Harlow because they were treated with TARGIT IORT (1 mile=1.61 km).

In the past 15 months (July 2014 to September 2015), the first 22 patients who received TARGIT IORT in Swindon (n=7) and Harlow (n=15) saved, on average, 753 miles (median 717, range 129–1751 miles; 1212 km (median 1154, range 208–2818 km)) of travel. Patients treated in Swindon would have travelled farther for EBRT than those in Harlow (1014 (SE 224) vs 631 (SE 130) miles per patient (1632 (SE 361) vs 1016 (SE 209) kilometres per patient); figure 4). These 22 patients would have driven a total distance of 16 572 miles (26 664 km) if they had received traditional EBRT. A total of 4.73 tonnes of CO_2_ would have been produced by these car journeys (215 kg/ patient). Each Swindon patient saved approximately 30.9 h (SE 3.3) of travelling time with mean journey times of 1 h 50 min each day (median 1 h 52 min, range 1 h 4 min–2 h 28 min). Harlow patients saved 18.5 h (SE 1.9) of journey times with mean daily return journey times of 1 h 6 min (median 19 h, range 28 min–1 h 38 min).

## Discussion

We found that within the TARGIT-A trial, UK patients in the prepathology stratum randomised to TARGIT saved themselves from travelling 305 miles (491 km) for 11 h and avoided CO_2_ emissions of 86 Kg. In the context of routine clinical practice in two hospitals outside of London, the saving per patient was much larger in terms of distance: 753 miles (1212 km), CO_2_ emissions—215 kg CO_2_ and time—between 19 and 31 h. Of course, we have not taken into account the actual psychological distress that may also be reduced.

This study is a detailed analysis of the distance travelled by UK patients within a large randomised international study. Use of the patient's postcode allowed accurate journey distances to be calculated and, by using UK-wide data from patients treated in four geographical centres, this allowed for a comparison of the travel implications for patients within an urban or semirural setting. The predicted travel times according to Google Maps were very close to the measured index journeys taken by the author. However, these journeys are likely to be an underestimate of actual times taken by patients as these cannot take into account any delays due to traffic or parking problems.

The medical literature contains few studies about the implications for patients and their families in travelling to receive radiotherapy. Our data are consistent with the one UK published study that showed that some patients were travelling up to 60 miles in each direction.^1^

### Estimation of impact on UK patients with breast cancer

In the UK, over 50 000 new breast cancers are diagnosed each year,^20^ of which approximately 75% receive breast-conserving surgery^21^ ^22^ and, depending on the definition of suitability of patients for IORT as a treatment modality (ASTRO, ESTRO or TARGIT criteria^23–25^), 15.8%, 34.1% or 54% of these patients could be offered single-dose TARGIT IORT. If this treatment was offered in the UK and established patient selection criteria applied, either 5925 12 800 or 20 250 may be saved many weeks of travel to receive EBRT. Using the very conservative estimate of travelling distance (provided by the TARGIT study patients), we estimate that this could save 1.8, 3.9 or 6.2 million miles of journeys and reduce UK CO_2_ emissions by between 516 and 1763 tonnes annually (table 1). It seems to us that a conservative estimate is 5 million miles of journey saved per year, that is, about 100 miles per breast cancer case diagnosed.

**Table 1:** Estimate of annual number of UK patients with breast cancer suitable for IORT, travel distances saved and reduction in CO_2_ emissions (1 mile=1.61 km)

Selection criteria	Proportion of UK patients (%)	Annual UK patients suitable for TARGIT-Alone	Travel distance saved (million miles) 305 mile/pt* or 753 miles/pt†	CO_2_ emissions saved (tonnes)	Area of forest to sequester annual CO_2_ emissions‡ (ha)
ASTRO§	15.8	5925	1.81*	516*	46.9*
			4.46†	1274†	115.7†
ESTRO§	34.1	12 800	3.90*	1115*	101.2*
			9.64†	2752†	249.9†
TARGIT¶	54	20 250	6.18*	1763*	160.2*
			15.25†	4353†	395.4†

*Estimate as per TARGIT-A trial patients.

†Estimate as per Swindon and Harlow patients.

‡Calculated using a value of three tonnes carbon sequestered per hectare per annum.§Based on consensus opinion only.^23^ ^24^

¶Based on randomised evidence (TARGIT-A trial) and two large cohort studies (French and German).^17^ ^25^

IORT, intraoperative radiotherapy.

It should be noted that early in the course of the TARGIT-A trial, most UK radiotherapy departments began to adopt the 3-week regimes rather than 6-week regimes, so the average number of fractions of radiation received by the trial patients was 17.5. The current provision of radiotherapy is concentrated in the larger hospitals in urban centres, so patients could face either a daily prolonged ‘cross-city commute’ or a longer journey from surrounding towns or villages. The geographical location of many moderate-sized UK breast centres is such that patients need to travel considerable distances to receive radiotherapy, and we need to remember that the potential environmental and economic savings of introducing TARGIT may be greater when applied to a breast cancer population outside of large UK urban centres.

These figures are likely to be an underestimate of the CO_2_ reductions as applied in the UK, as we have not taken into account traffic conditions. Similarly, the savings in travel times, mileage and CO_2_ for patients is likely to be even greater as many UK breast units do not have an on-site radiotherapy centre, as is the case with two of the six TARGIT-A trial centres (Whittington and Winchester) that required their patients to travel to a different hospital for EBRT. Typically, most UK patients, just as the patients treated in Swindon or Harlow, would thus travel more than twice the distance of their TARGIT study counterparts (753 vs 305 miles; 1212 vs 491 km).

Carbon sequestration by woodland is often promoted to offset carbon production. Depending on tree species, the annual rate of carbon absorption for UK forests is 2–5 tonnes/hectare.^26^ One tonne of carbon is contained within 3.67 tonnes of CO_2._ Using a figure of 3 tonnes/hectare per year, we estimate that failing to introduce TARGIT in the UK would require continuous maintenance of an area of mature forest over an area of 47–395 hectares to offset the CO_2_ produced by travel of these selected patients to receive radiotherapy. This forest would need to cover St Jame's Park (23ha), Green Park (19ha), Buckingham Palace Gardens (17ha), Kensington Gardens (111Ha) and Hyde Park (142ha) combined.

The use of public transportation or bicycles and walking, particularly in London, may change these calculations of the carbon footprint. However, the vast majority of patients in the rest of the country would have used a car for which these calculations are valid.

As clinicians, we need to remember the impact of our prescribed therapies on patients and their relatives. In rural areas, chemotherapy services may be provided closer to the patient's home to reduce the need to travel, and indeed, breast screening is usually available using mobile units. However, the need to travel to receive radiotherapy has an environmental impact that, until now, has been ignored. We have demonstrated that providing single-dose TARGIT treatment to selected UK patients with breast cancer will benefit patient travel times and CO_2_ emissions. Given that patients with breast cancer constitute about a third of patients in a radiotherapy department, this should also reduce the traffic congestion around the hospital.

This is the first study to quantify the environmental benefit of introducing TARGIT IORT and demonstrates the magnitude of the impact on our environment of asking our patients to travel to receive centralised radiotherapy services. The analysis and concepts described are applicable in every aspect of healthcare where a patient is required to travel to receive a series of treatments. While the term ‘food-miles’ has become commonplace in the mindset of the general public, perhaps the concept of ‘therapy-miles’ ought to be considered when planning and prescribing patient treatment.

Our finding of a saving of about 87 to 215 kg CO_2_ emissions for each patient receiving TARGIT (and thus from making TARGIT available to every patient with breast cancer having a lumpectomy in the NHS) is considerably greater than the estimated savings of the study investigating the benefits of introducing a different mode of improving access to services, namely, mobile breast screening units, which is about 1.25 kg CO_2_ per woman screened.^27^

We conclude that introducing TARGIT as an option for appropriate patients in the UK will contribute significantly to saving patients time, cost, fuel and CO_2_ emissions.
