# Supplementary material for: Environmental and social benefits of the targeted intraoperative radiotherapy for breast cancer: data from UK TARGIT-A trial centres and two UK NHS hospitals offering TARGIT IORT
Source: BMJ Open. 2016 May 3;6(5):e010703. doi: 10.1136/bmjopen-2015-010703 (PMC4890331; doi:10.1136/bmjopen-2015-010703)
Supplement: Supplementary tables [file bmjopen-2015-010703supp_tables.pdf]

Supplementary Table 1: Measured and estimated distances and one-way journey times from Swindon to local Radiotherapy Centres (1 mile = 1.61 km)

**Destination Radiotherapy Centre**	**Number of Journeys tested**	**Measured mean distance driven from Swindon (miles) and range**	**Google Maps® estimate of one-way travel distance (miles)**	**Measured mean one-way journey times (minutes) and range**	**Google Maps® estimate of one-way journey time (minutes)**
Oxford	8	36.6 (36.4 - 36.8)	36.4	53 (44 - 62)	52
Bath	4	39.0 (38.4 - 39.6)	38.6	47 (44 - 50)	45

Supplementary Table 2: UK Population living within cities or towns containing a radiotherapy centre (UK Office for National Statistics 2011) (Office for National Statistics, 2011) [28]

City/ Town with RT Centre	Population	
London	8,600,000	
Newcastle	1,650,000	
Birmingham	1,085,400	
Leeds	751,500	
Glasgow	592,820	
Belfast	579,276	
Sheffield	551,800	
Manchester	510,700	
Leicester	509,000	
Edinburgh	486,120	
Liverpool	465,700	
Bristol	437,500	
Cardiff	346,000	
Coventry	316,900	
Nottingham	310,837	
Brighton	273,369	
Hull	256,406	
Plymouth	256,400	
Southampton	253,651	
Wolverhampton	249,470	
Derby	248,752	
Swansea	239,000	
Romford	237,232	
Aberdeen	217,120	
Norwich	213,166	
Northampton	212,000	
Portsmouth	209,166	
Peterborough	183,600	
Colchester	176,008	
Southend	174,800	
Reading	155,698	
Canterbury	151,200	
Oxford	150,200	
Poole	149,000	
Dundee	144,290	
Middlesborough	138,400	
Guildford	137,200	
Preston	135,100	
Ipswich	133,400	
Lincoln	132,500	
Stafford	130,800	
Cambridge	123,867	
Exeter	117,773	
Cheltenham	115,700	
Taunton	110,200	
Carlisle	107,500	
Basingstoke	107,355	
Shrewsbury	102,382	
Londonderry	90,736	
Bath	88,859	
Torquay	65,245	
Inverness	56,600	
Truro	20,332	
**Population within cities or towns with RT centres**	**23,258,030**	(37%)
**Total UK population**	**63,182,000**	
**Population outside of cities or towns with RT centres**	**39,923,970**	(63%)
